# Effect of Tai Chi for post-stroke mental disorders and sleep disorders: A protocol for systematic review and meta-analysis

**DOI:** 10.1097/MD.0000000000012554

**Published:** 2018-09-28

**Authors:** Fan Yang, Diyang Lyu, Ruyu Yan, Yahui Wang, Zongheng Li, Yihuai Zou, Yong Zhang

**Affiliations:** a Department of Rehabilitation; b Department of Neurology and Stroke Center, Dongzhimen Hospital, The First Affiliated Hospital of Beijing University of Chinese Medicine, Beijing, China.

**Keywords:** mental disorders, meta-analysis, sleep disorders, stroke, systematic review, Tai Chi

## Abstract

**Background:**

Post-stroke mental disorders (PSMDs) and post-stroke sleep disorders (PSSDs) are very common in stroke patients. Recently, Tai Chi (TC) as a form of Chinese traditional mind-body exercise has been gradually applied to stroke rehabilitation although its efficacy for PSMD and PSSD varies across different studies. The aim of this study is to explore the therapeutic effect of TC training for PSMD and PSSD.

**Methods:**

This review will only include randomized controlled trials (RCTs). Search strategy will be performed in 3 English databases, 4 Chinese databases, and the WHO International Clinical Trials Registry Platform. All English or Chinese RCTs, published from inception to February 28, 2019, will be sought. Two reviewers will screen, select studies, extract data, and assess quality independently. Primary outcomes are clinical scales, mainly including “Hamilton depression scale,” “Hamilton anxiety scale,” and “Pittsburgh sleep quality index.” The methodological quality including the risk of bias of the included studies will be evaluated using a modified assessment form, which is based on Cochrane assessment tool and Physiotherapy Evidence Database scale. Review Manager Software (*Revman5.3*) will be used for heterogeneity assessment, generating funnel-plots, data synthesis, subgroup analysis, and sensitivity analysis. We will use GRADE system to evaluate the quality of our evidence.

**Results:**

We will provide some more practical and targeted results investigating the effect of TC exercise for PSMD and PSSD in the current meta-analysis. Meanwhile, we will ascertain study progress of TC for PSMD and PSSD and find out defects or inadequacies of previous studies, so that future researchers could get beneficial guidance for more rigorous study.

**Conclusion:**

The stronger evidence about TC's rehabilitative effect and safety for PSMD and PSSD will be provided for clinicians and policymakers.

**Systematic review registration:**

PROSPERO CRD42018106608.

**Ethics and dissemination:**

We do not apply for formal ethical approval from ethics committee because all of the study data in our review will be obtained in an anonymous way. Findings of this study are projected to be disseminated through peer-review publications.

## 1 Introduction

### 1.1 Description of the condition

Stroke is the second greatest cause of death and one of the leading causes of disability,^[1]^ and this condition may continue until 2030.^[2]^ In the past decades, the number of new-onset stroke people every year and stroke survivors have been great and increasing,^[3]^ which leads to a huge challenge for governments, especially those in low-income and middle-income countries, because of the large medical burden. With the highest rates of stroke mortality and incidence, China bears the heaviest stroke burden in the world.^[4]^ Mental and sleep disorders, such as depression, anxiety, phobic disorders, insomnia, and so on, exist widely among stroke patients, because various factors have notably negative effect on their spiritual status.^[5–7]^ According to some retrospective literature reported over years, the frequency of anxiety and depression after stroke is approximately 25%^[8]^ and 31%,^[9]^ respectively. Quite a few stroke patients (21.3% and 39.0%, n = 282) experience less night sleep duration and more daytime sleepiness than before stroke.^[10]^ Moreover, apathy^[11]^ and social inactivity^[12]^ are not rare in stroke adults. The presence of above mental disorders may result in not only delayed functional recovery^[13,14]^ and decline in quality of life,^[15,16]^ but also higher risk for stroke recurrence,^[17]^ increased mortality, and cognitive impairment.^[18]^ So the enhancing of recognition and management for post-stroke mental disorder (PSMD) and post-stroke sleep disorder (PSSD) is absolutely necessary. However, to our regret, the current concern for this issue is relatively scarce because the most common functional deficit is motion disability, which is easier to get attention from clinicians or rehabilitation therapists.

### 1.2 Description of intervention

Tai Chi (TC), also named Tai Ji or Tai Ji Chuan, is originated from ancient Chinese martial art. As a form of the traditional mind-body exercise with a long history, TC exercise has become increasing popular worldwide recently.^[19]^ It's widely practiced among the normal,^[20,21]^ as well as among patients with cardiac,^[22,23]^ pulmonary,^[24]^ orthopedic,^[25,26]^ endocrine,^[27,28]^ neurological,^[29]^ rheumatic^[30,31]^ diseases, and even cancer,^[32]^ because its beneficial effects have been validated on corresponding conditions. In addition to the physical diseases, previous studies paid attention to the mental disorders. For example, TC training was found to be associated with reduced cancer-related depression,^[33]^ relieved cancer-related sleep difficulty,^[33]^ and lower level of anxiety^[23]^ among patients with coronary heart disease. Although there are increasing studies tend to focus on TC's effect for post-stroke mental complications, their conclusions are not absolutely consistent. Therefore, systematic reviewing the efficacy of TC training for PSMD and PSSD, by which we can provide further evidence for clinicians and policymakers, becomes very essential and meaningful.

### 1.3 Objective of this study

To conduct a systematic review and meta-analysis determining the therapeutic efficacy of TC practice for PSMD and PSSD.

## 2 Methods

This review protocol is registered in the PROSPERO International Prospective Register of systematic reviews, registration number CRD42018106608. Cochrane Handbook for Systematic Reviews of Interventions (Version 5.1.0, http://www.cochrane-handbook.org) will be used as guidance to conduct this systematic review. The statement of preferred reporting items for systematic review and meta-analysis protocols ^[34]^ and preferred reporting items for systematic reviews and meta-analyses (PRISMA)^[35]^ will serve as guidelines for reporting present review protocol and subsequent formal paper.

### 2.1 Inclusion criteria for study selection

#### 2.1.1 Types of studies

We will only include randomized controlled trials (RCTs), whereas non-RCTs, quasi-RCTs, and any other types of studies will be excluded.

#### 2.1.2 Types of participants

Referring to the World Health Organization's definition^[36]^ of stroke, all ischemic or hemorrhagic adult stroke patients regardless of race, region, sex, and the phase of stroke will be included. All the participants must be made a definite diagnosis of stroke by brain computed tomography or magnetic resonance imaging.

#### 2.1.3 Types of interventions

As we know, TC contains multiple styles because of long process of development and evolution. It's hard to realize standardization or unification in different TC studies. Thus, we will accept all types of TC interventions without any restrictions of their style, training mode, duration, and frequency. The treatment group should be treated by TC combining or not combining with conventional rehabilitative treatment, whereas the control group should receive conventional rehabilitation therapies, which is the same with that in treatment group, or receive other forms of exercises or nothing at all. However, if the treatment group receive 2 or more forms of exercises, such as TC combined with Yoga or others, we will decide whether to incorporate it into analysis depending on its control group set. Subgroup analysis will be performed according to above factors to obtain more objective results. Some other cointervention programs for stroke, such as routine medication, lifestyle modifications, and health education, are acceptable, as long as they are equally applied to both the treatment and control group.

#### 2.1.4 Types of outcome assessments

##### 2.1.4.1 Primary outcomes

The rehabilitative effect of TC training for PSMD and PSSD is mainly evaluated by the pre-post score changes of different clinical scales. Thus, we will adopt some internationally recognized scales as the primary outcomes, including “Hamilton depression scale,” “Hamilton anxiety scale,” “the mental health part of the MOS item short from health survey (SF-36),” “Generic Quality of Life Inventory-74,” “Center for Epidemiologic Studies Depression Scale,” and “Pittsburgh sleep quality index.”

##### 2.1.4.2 Secondary outcomes

Secondary outcomes mainly involve adherence to TC exercise, adverse events, and all-cause death during the entire treatment and follow-up period.

#### 2.1.5 Search strategy

To avoid missing any available literatures, which possibly meet our demands, we will systematically search following electronic databases: MEDLINE, EMBASE, The Cochrane Library, the Chinese National Knowledge Infrastructure, the Chinese BioMedical Literature Database, the Chinese Science and Technology Periodical Database, and Wanfang. All the English and Chinese literatures, published from inception to February 28, 2019, will be sought without the limitation of race, sex, and region. Meanwhile, our search will also cover the WHO International Clinical Trials Registry Platform and its Registry Network to gain additional studies unpublished or being prepared for publication. In addition, the reference lists of previous clinical studies and reviews will be served as the searching object. Search strategy will be built according to guidance from the Cochrane handbook. Search strategy for EMBASE is shown in Table 1, and similar strategies will be built and applied for other electronic databases.

**Table 1:**
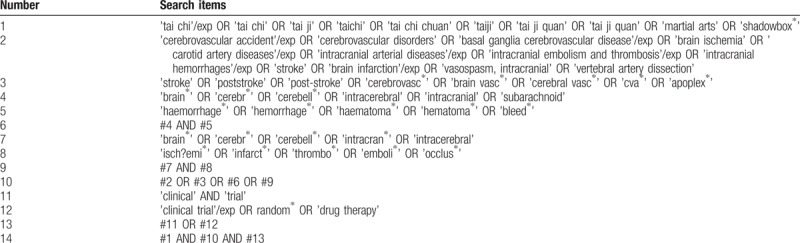
Search strategy for EMBASE.

### 2.2 Data collection and analysis

#### 2.2.1 Selection of studies

**Figure 1:**
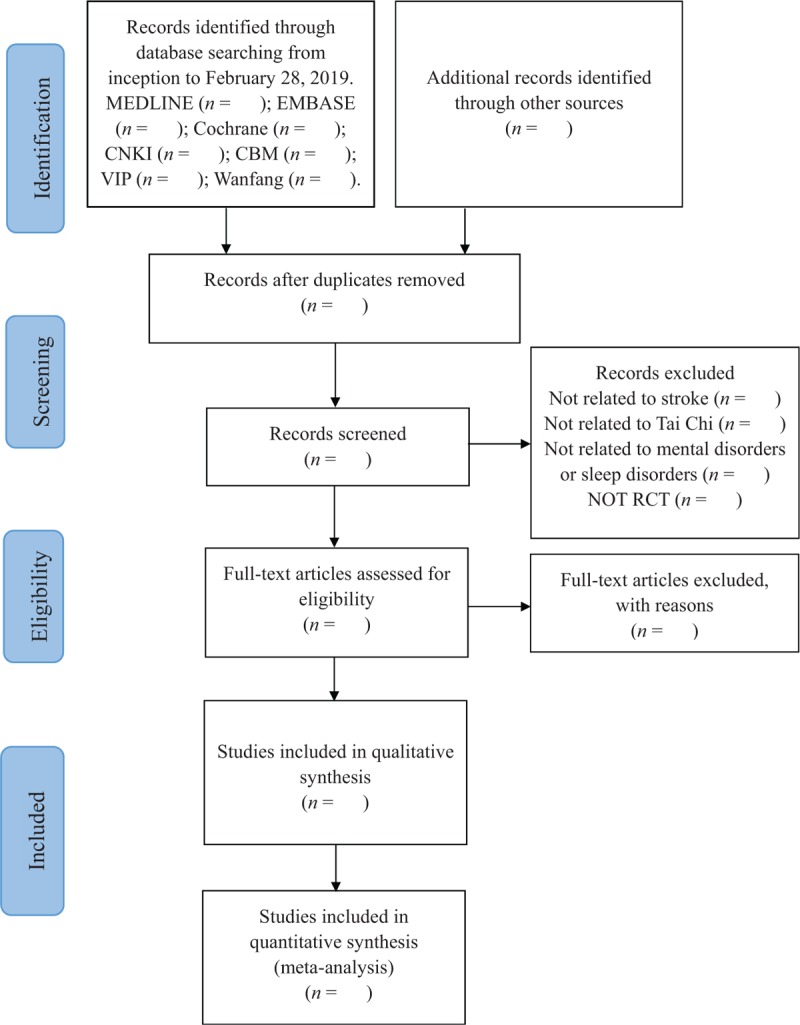
Flow diagram of study selection. CBM = Chinese BioMedical Literature Database, CNKI = Chinese National Knowledge Infrastructure, VIP = Chinese Science and Technology Periodical Database, RCT = randomized controlled trial.

Firstly, 2 review authors (FY and RY) will independently check the titles and abstracts of retrieved results and preliminarily select potentially suitable articles. *Endnote X7* software will be used to record and manage them. Secondly, through seriatim reading full texts of preliminary selective articles, 2 independent reviewers select eligible studies according to our predetermined inclusion criteria. Finally, articles selected by 2 independent reviewers will be put together after eliminating the identical. If 2 articles represent the duplicate publications of 1 study, only the 1 with the fullest data will be included. To resolve disagreements regarding inclusion or exclusion, 2 independent reviewers will first discuss with each other, and then make a consultation with another experienced reviewer (YZ). All eligible studies will be included for qualitative and (or) quantitative analysis. Details of the entire selection procedure will be shown in a PRISMA flow chart^[37]^ (Fig. 1).

#### 2.2.2 Data and information extraction

We will make a detailed data and information extraction form (Table 2) consisted mainly of following items:

1. Publication information (Name of first author, Contact details, Year, Country and region);
2. Participants characteristics (Source, Sample size, Average age, Sex ratio, Stroke duration, Stroke types and severity, Lesion side, Compliances of mental disorders or sleep disorders, Usage of antipsychotics or drug for sleep disorders);
3. Interventions (TC styles, Training frequencies and training time of every time, Total training time);
4. Comparison (Treatment ways and types, Frequencies, Treatment time or dose of every time, Course of treatment);
5. Outcomes and others (Scale instruments, Assessment time, Details of results, Informed consent, Drop-out rate and reasons, Adverse events, Costs and funding sources);
6. Study design (Randomization, Blinding).

**Table 2:**
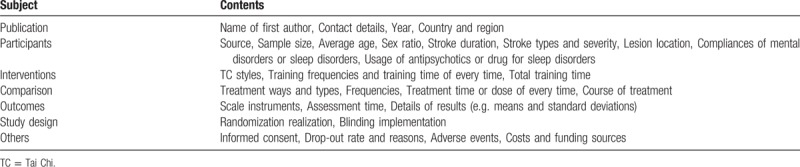
Data and information extraction schedule.

All above information or data will be obtained through reading the full text and confirmed by contacting original investigators. *Microsoft Excel 2013* will be used for data and information management.

#### 2.2.3 Dealing with missing data

The missing data may influence study result to a certain degree, or even lead to different study conclusion. So in the process of data extraction, we will contact article authors or original investigators to make sure whether there exists missing data in every single included study. If there exist missing data, we will further check and record how they dealt with it when they made a statistical analysis, and assess whether their processing methods are reasonable. If there is no possibility for the processing approaches to significantly distort their statistical results, we will make their data merged. Otherwise, we will have to quit synthesize those data to reduce bias. For a small amount of study results lacking standard deviations, we will attempt to get from original investigators. If the attempt fails, we will try to repair them through borrowing standard deviations from a most similar study. It matters that we will analyze and report the potential impact of missing or incomplete data for the aggregated result.

#### 2.2.4 Appraisal of study quality

**Figure 2:**
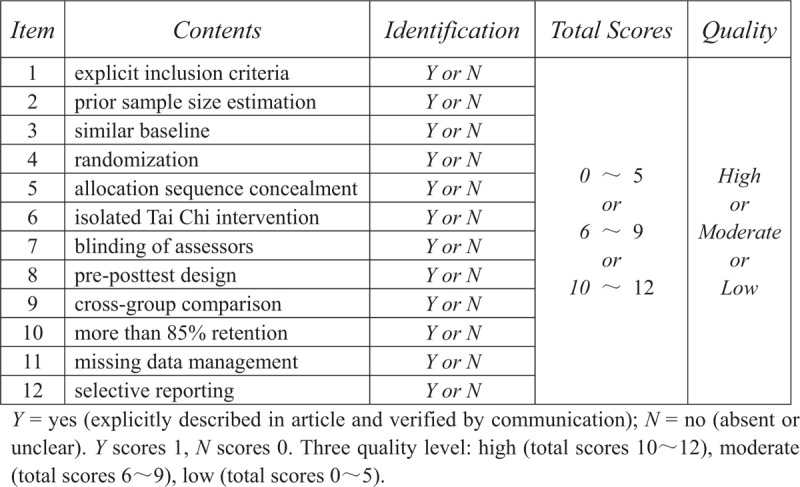
Modified assessment form. *Y* = yes (explicitly described in article and verified by communication); *N* = no (absent or unclear). *Y* scores 1, *N* scores 0. Three quality level: high (total scores 10–12), moderate (total scores 6–9), low (total scores 0–5).

Given to the specificity of TC intervention, we make a modified assessment form, which is based on Cochrane assessment tool for risk of bias and Physiotherapy Evidence Database (PEDro) scale, to evaluate methodological quality of eligible studies. This modified assessment form mainly contains following 11 items: item 1 = explicit inclusion criteria; item 2 = prior sample size estimation; item 3 = similar baseline; item 4 = randomization; item 5 = allocation sequence concealment; item 6 = isolated TC intervention; item 7 = blinding of assessors; item 8 = pre-posttest design; item 9 = cross-group comparison; item 10 = more than 85% retention; item 11 = missing data management (if missing data are present); item 12 = selective reporting. Every item will be rated as *Y* = yes (explicitly described in article and verified by communication), or *N* = no (absent or unclear). The item identified *Y* scores 1, and the item identified *N* scores 0. According to different total points, every study will be categorized into 3 quality level: high (10–12), moderate (6–9), and low (0–5). Details of the qualitative assessment are shown in Figure 2.

Before the identification of above methodological items, 2 independent reviewers (FY and RY) will make beforehand communication and verification with original authors to avoid misjudgment. As a primary basis for evaluating study quality and making classification, all the replies or explanations from original authors will be recorded in detail. Any divergences will be resolved through discussion and consultation with a third experienced reviewer (YZ).

#### 2.2.5 Assessment of reporting bias

We will generate funnel plots to assess reporting bias if no <10 studies are available for quantitative analysis. For continuous variables, Egger test will also be taken to test funnel plot asymmetry. However, even when a test does not provide evidence of funnel plot asymmetry, reporting bias (including publication bias) cannot be excluded due to the relatively low test power. Asymmetric funnel plots are usually thought to present publication bias, one type of the reporting biases, but it also means that there may exist other reasons, such as differences in methodological quality or true heterogeneity in intervention effects. We will analyze the possible cause and give reasonable interpretation for asymmetric funnel plots.

#### 2.2.6 Assessment of heterogeneity

Assessment of heterogeneity involves 2 heterogeneity tests, χ^2^ test (significance level: 0.1) and *I*^2^ test. The previous one checks whether there exists heterogeneity, and the latter reflects the degree of heterogeneity through a specific value (usually 25% or lower = low, 25% to 75% = medium, 75% or more = high). We will analyze the possible sources of high heterogeneity when it emerges.

#### 2.2.7 Measure of treatment effect

For dichotomous variables, such as adverse event, we will calculate risk ratio or odds ratio with 95% confidence interval (CI). For continuous variables, mean difference or standard mean difference with 95% CI will be calculated.

#### 2.2.8 Data synthesis

Quantitative synthesis will be done after qualitative analysis. Eligible studies with complete data and without missing data will be quantitatively synthesized. Studies with incomplete data will also be included for quantitative synthesis on condition that their data could be retrieved or reasonably repaired. Studies with incomplete data all along and (or) with unreasonable processing approaches for missing data will only be qualitatively analyzed. Review Manager Software (*Revman5.3*, available from the Cochrane Web site http://tech.cochrane.org/revman) will be used to carry out quantitative data synthesis. If the *I*^2^ value is no >50%, which indicates relative smaller heterogeneity, the fix-effect model shall be employed to obtain synthesis results. Otherwise, the random-effect model will be adopted.

#### 2.2.9 Subgroup analysis

Allowing for the possibility of high heterogeneity, we will make a subgroup analysis project to obtain an objective conclusion. Firstly, data from participants in different recovery stages (within 1, 2–6, and beyond 6 months) will be separately analyzed. Secondly, data based on different comparative designs, such as TC vs blank control, TC vs conventional rehabilitation treatment (CRT), and TC combined with CRT vs CRT, will be separately analyzed. Thirdly, if possible, data according to varied TC styles, training time, and frequencies will be respectively analyzed. In addition, some factors including methodological quality of trials, age, lesion location or nature, severity, and prior mental or sleep disorder may lead to high heterogeneity; subgroup analysis need take these into account.

#### 2.2.10 Sensitivity analysis

After the data synthesis, we plan to conduct sensitivity analysis through excluding merged studies one by one and observe whether the synthesis result changes significantly. Significant change reflects that the removing study is enough to influence overall synthesized result, so, it is necessary for us to reassess it and decide cautiously whether to merge it. A valid reason must be given before we make a decision. If no significant change arises, we could consider that our synthesized result is firm.

#### 2.2.11 Quality of evidence

Internationally accredited GRADE system will be used to evaluate the quality of our evidence. We will apply *GRADEpro3.6* software to make qualitative assessment of evidence level. Considering the fact that only RCTs are accepted, we will perform a downgrade quality of evidence mode which involves the following 5 factors: risk of bias, inconsistence, indirectness, imprecision, and publication bias. The level of evidence will be graded as high, moderate, low, and very low.

## 3 Discussion

Some mind-body exercises, such as TC, Qigong, and Yoga, are often recommended to regulate mood or emotion for stroke patients. However, few high-quality studies could provide strong evidence about their efficacy and safety. Investigators have made some systematic reviews or meta-analyses to get comprehensive evidence in recent years. A meta-analysis demonstrated that TC exerts beneficial effects on depression, anxiety, and stress management for various populations.^[38]^ Another meta-analysis showed that TC training may improve sleep quality of the old.^[39]^ A meta-analysis based on various populations (healthy or sick) indicated that Qigong may reduce the severity of depressive symptom, whereas TC may not, even if both of them are mind-body movement.^[40]^ Nevertheless, all of the above conclusions are not drawn within stroke patients. The latest meta-analysis determining the effect of mind-body exercises for stroke patients’ mood found that mind-body exercises have benefits on reducing depression and anxiety, but they may not improve sleep quality.^[41]^ It is noteworthy that this study lacks subgroup analysis according to the types of different mind-body exercises, which may lead to relative broad conclusions. To the best of our knowledge, there has been no one meta-analysis specially analyzing TC's effect for PSMD and PSSD. We hope to provide more practical and targeted results investigating the effect of TC exercise for PSMD and PSSD in the current systematic review and meta-analysis.

As is known, the key to achieve a reliable meta-analysis result lies in incorporating sufficient data from high-quality original literature and perform rigorous methodological quality assessment. Allowing for the particularity of TC, we make a modified assessment form which incorporates the advantages of Cochrane assessment tool and PEDro scale, making our qualitative evaluation more reasonable and practical. And also, it is sensible that our quality assessment will not only include reading original articles to know methodological execution but also making verification with original authors to reduce the possibility of misjudgment.

The strengths of our study mainly include that comprehensive searching for Chinese and English databases, rigorous evaluation of quality, and sensible subgroup analysis design, all of which will make our analysis result more convictive. One limitation of this review is that we will only search Chinese and English databases, possibly missing some articles published using other language. Another limitation is that the large heterogeneity may emerge, leading to adverse effect on the final conclusion.

## Author contributions

Fan Yang, Diyang Lyu, and Yong Zhang conceived the study. The protocol was drafted by Fan Yang and Diyang Lyu, and revised by Yihuai Zou and Zongheng Li. Both Fan Yang and Diyang Lyu serve as the first author. Diyang Lyu and Yahui Wang developed the search strategy. Fan Yang and Ruyu Yan will independently work on study selection, quality assessment, data extraction, and synthesis.

**Conceptualization:** Fan Yang, Zongheng Li, Yihuai Zou, Yong Zhang.

**Data curation:** Ruyu Yan, Yahui Wang.

**Formal analysis:** Fan Yang.

**Funding acquisition:** Zongheng Li, Yong Zhang.

**Investigation:** Ruyu Yan, Yahui Wang.

**Methodology:** Fan Yang, Diyang Lyu, Yong Zhang.

**Software:** Diyang Lyu, Yahui Wang.

**Supervision:** Yong Zhang.

**Writing – original draft:** Fan Yang, Diyang Lyu.

**Writing – review and editing:** Zongheng Li, Yihuai Zou.

Fan Yang orcid: 0000-0002-9083-3035
